# DNA Condensates Enable Crosstalk‐Free Operation of Identical DNA Computing Cascades

**DOI:** 10.1002/anie.5954994

**Published:** 2026-04-20

**Authors:** Weixiang Chen, Rahmetullah Demirci, Miao Xie, Andreas Walther

**Affiliations:** ^1^ Life‐Like Materials and Systems Department of Chemistry University of Mainz Mainz Germany; ^2^ Max Planck Institute for Polymer Research Mainz Germany

**Keywords:** DNA computing, DNA condensates, DNA nanotechnology, supramolecular chemistry, systems chemistry

## Abstract

DNA strand displacement reactions (SDR) have enabled the development of biosensing devices, molecular machines, and molecular computing. However, the need for high sequence orthogonality poses a major challenge to the modular integration and scaling of DNA SDR networks into more complex systems capable of advanced or parallel functions. Here, we propose the use of liquid‐like DNA condensates with addressable barcodes for confining DNA SDR networks for parallel and selective operation of near‐identical circuits that would otherwise show undesired crosstalk in a homogeneous solution. By introducing Transducer modules, specific inputs can be recognized by corresponding condensates and converted to a unified Messenger for triggering downstream DNA processing locally. This allows orthogonal execution of DNA SDRs of the same sequence design in different compartments in parallel without crosstalk and interference. Our strategy contributes a facile approach to enhance modularity and scalability in DNA SDR network design, paving the way for more sophisticated and complex functionalities.

## Graphical abstract

DNA condensates have been developed and employed in dynamic DNA nanotechnology to provide compartmentalization for DNA computing cascades. This versatile strategy enables parallel, crosstalk‐free DNA reactions among systems with identical sequence designs, demonstrating a high degree of orthogonality.

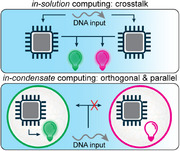

## 1 Introduction

Compartmentalization is an important concept at every level of living systems that possess complex functions [1, 2, 3]. Even in the basic units of life, for example, a eukaryotic cell, functions and processes are organized in space using sub‐cellular structures such as phase‐separated organelles, mitochondria, and the endoplasmic reticulum [2, 4, 5]. This allows competing or interfering processes to operate more efficiently or to operate at all in parallel.

The advent of DNA nanoscience has given rise to the programmable construction of nanostructures, switchable nanodevices, as well as to information processing through DNA‐based reaction networks (DNA‐RNs) [6, 7, 8, 9, 10]. Among the latter, toehold‐mediated DNA strand displacement reactions (SDR) have enabled concepts for disease diagnostics, switchable materials and self‐assemblies, as well as computation [11, 12, 13, 14, 15, 16, 17, 18, 19]. However, two major challenges presently constrain further development of DNA‐RNs: Sequence overlap and interference limit the scale and complexity of larger systems [20]. The modular integration of different DNA‐RNs into a single system is challenging because it often requires major changes or even a complete redesign of the sequences. For example, in a recent study reporting a general‐purpose DNA computation system consisting of hundreds of DNA strands, the DNA‐RN had to be executed in individual subunits of selected DNA gates, separated in different vials [21]. Signal transduction between individual parts of the DNA‐RNs required additional steps, including DNA registration on a DNA origami scaffold and manual purification and transfer. Overcoming these issues requires establishing a robust compartmentalization strategy to identify approaches for running otherwise interfering DNA‐RNs in compartmentalized objects that have access to a global input‐output space, but prevent unwanted crosstalk. In this sense, parallelized computing in water‐in‐oil droplets would not be useful, as the water droplets cannot access the same input‐output space. Indeed, there have been a few attempts using DNA origami to physically separate DNA‐RNs, demonstrating the possible reusability of the same sequence design for different DNA‐RNs in the same solution [22, 23]. However, a DNA origami highly immobilizes DNA strands with little diffusivity, limiting at some point the development of more complex DNA‐RNs. There have been some reported studies using DNA condensates based on DNA nanostars to host DNA reactions, which have, however, not been leveraged to eliminate reaction crosstalk and interference [24, 25, 26]. A more recent study has successfully achieved local RNA transcription in such DNA condensates, which leads to a biased local SDR reaction. However, as there is no immobilization of transcribed RNA, the system ultimately leads to a global reaction with crosstalk at later stages [27].

Thinking about an ideal compartmentalization strategy for DNA‐RNs, the following criteria should be met: (1) Permeability and material exchange between inside and outside, for receiving signals to execute DNA‐RNs and release outputs. (2) Enrichment of desired DNA strands, rejection of undesired ones, and prevention of diffusive loss of important DNA strands, thus achieving high effective concentrations. (3) High mobility of DNA strands inside the compartments for a dynamic reaction. Here, we propose the use of liquid‐like DNA condensates as a powerful design for compartmentalizing DNA‐RNs. By physically isolating individual DNA‐RNs within distinct condensates, interference and crosstalk are efficiently suppressed, thereby eliminating the need for orthogonal sequence design. To address the condensates selectively in parallel, we design an input‐selective signal transduction mechanism that enables condensates to use the same DNA sequence design to respond to different DNA inputs.

## 2 Results and Discussion

**FIGURE 1:**
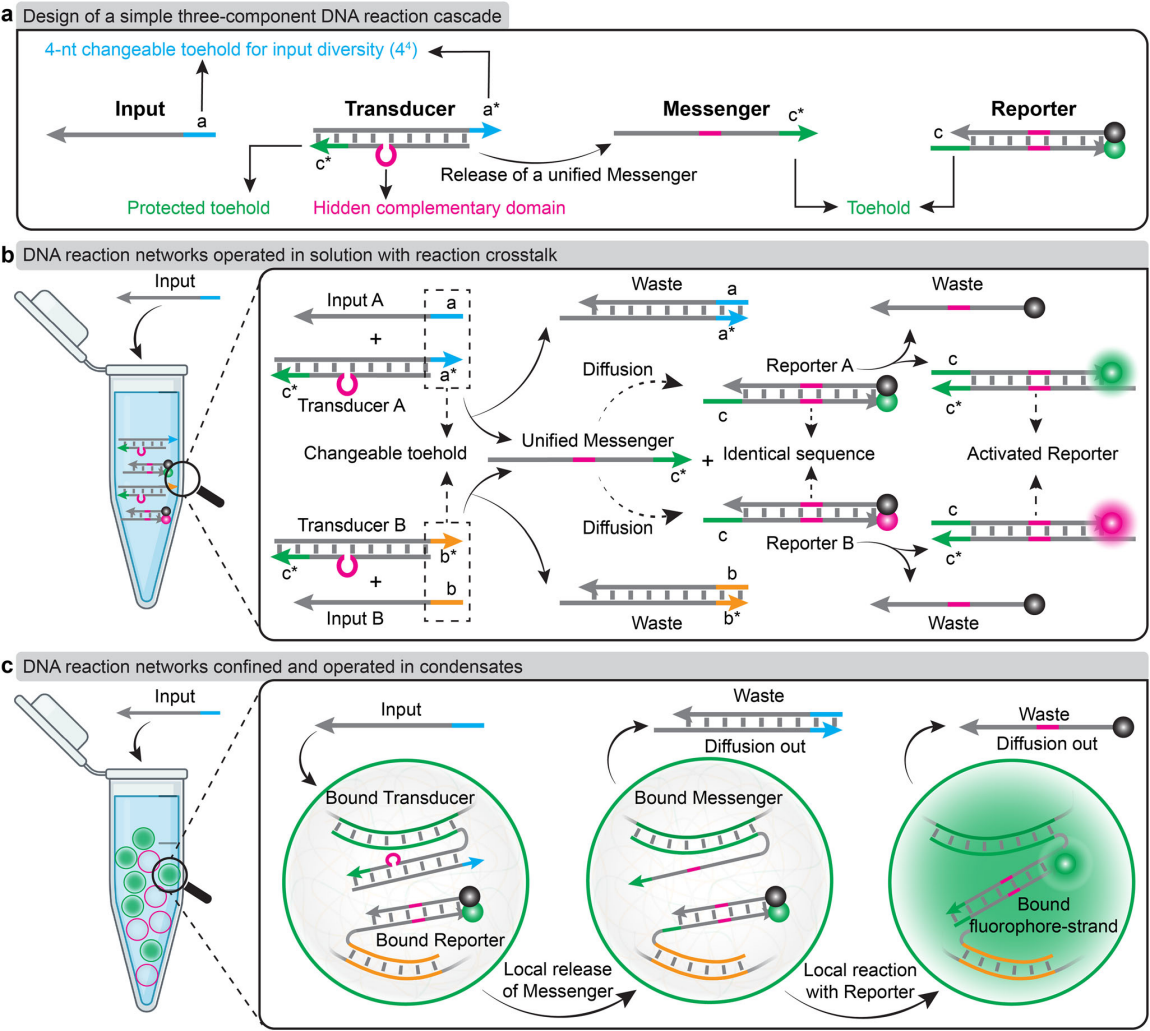
Design of a three‐component DNA reaction cascade and comparison of the reaction process in solution and in condensates. (a) Scheme showing the design of the three‐component system, including an Input, a Transducer, and a Reporter. The Input can react with the Transducer by toehold‐mediated SDR and release a Messenger, which can react with the Reporter. (b) Scheme of the DNA reactions in solution containing two sets of the three‐component system, where any of the two Inputs (Input A and Input B) reacts with its corresponding Transducer, which releases a unified Messenger for activating both Reporters A and B. (c) Scheme of the DNA reaction in condensates, where the Input reacts with the Transducer to locally release the Messenger, which activates the Reporter in the same condensate. Created with BioRender.com.

To exemplify the effect of compartmentalization on running interfering SDR circuits in parallel and orthogonal, we resort to simple three‐component systems composed of Inputs, Transducers, and Reporters (Figure 1a): We use two DNA reporter duplexes (Reporter A and Reporter B) that are identical in sequence but have different fluorophore‐quencher pairs. They can report a strand (Messenger) ejected by the Transducer. We use two Transducers that have distinct 4‐nt toeholds, but are otherwise identical and can eject an identical Messenger if the correct Input is added. This overall allows to engineer a sequence diversity of theoretically 4^4^ = 256 (in practice, it is less than 256 as there is sequence overlap in some sequence designs) distinct Input signals (Figure S1a,b for details of sequence design). Note that the Messenger has a non‐bonded loop region in the Transducer to ensure that the Inputs cannot directly react with the Reporters. It should be highlighted that this Input‐Transducer‐mediated Messenger release for simple Reporter activation can be further extended to trigger more complicated reaction networks. Several principles should be considered when compartmentalizing such a DNA reaction cascade inside DNA condensates, as further detailed in Figure S1.

Critically, when placing both Reporters and both Transducers into a bulk solution, there is unavoidable crosstalk, as either of the two Inputs can activate both Reporters, because diffusion happens globally and the Transducer produces a unified Messenger for both Inputs (Figure 1b). However, when compartmentalizing pairs of Reporter/Transducer orthogonally into condensates, the downstream SDR can be confined within each condensate because parts of the modules are tethered to the compartment (Figure 1c).

**FIGURE 2:**
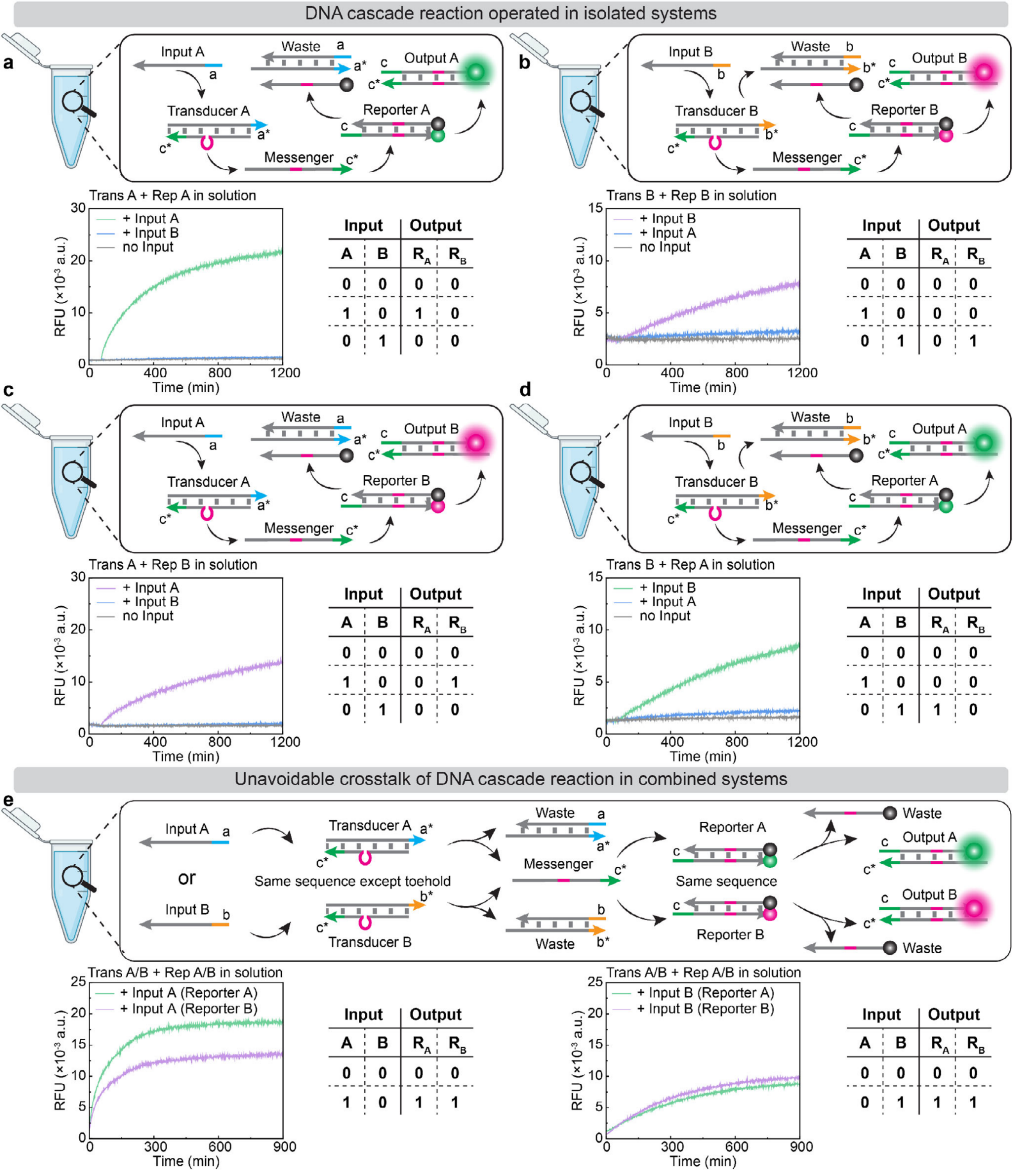
In‐solution DNA‐RNs with corresponding truth table show unavoidable reaction crosstalk in combined systems. (a) Input A reacts with Transducer A to release a Messenger, which activates Reporter A to produce a fluorescence signal. (b) Input B reacts with Transducer B to release a Messenger, which activates Reporter B to produce a fluorescence signal. (c) Input A reacts with Transducer A to release a Messenger, which activates Reporter B to produce a fluorescence signal. (d) Input B reacts with Transducer B to release a Messenger, which activates Reporter A to produce a fluorescence signal. (e) In case of Transducers A, B, and Reporters A, B in the same solution, any of the two Inputs A and B reacts with its corresponding Transducer to release a Messenger in the whole solution, which activates both Reporters A and B. R_A_: Reporter A; R_B_: Reporter B. Sample size (*n*) = 2. The curve represents the mean value. Created with BioRender.com.

We first analyze selectivity and crosstalk in solution in the absence of any condensates. Figure 2a–d demonstrates that the correct Input/Transducer combination enables activation of either of the two Reporters (See Figure S1c,d for a gel electrophoresis of an exemplified reaction for system A). For instance, Figure 2a,c displays activation of Reporter A and Reporter B, respectively, when Input A is combined with Transducer A. However, when Input B is added to the same system, there is no Reporter activation because Input B cannot react with Transducer A. No Input as a control leads equally to no reaction. Similarly, Figure 2b,d shows the inverted system, where Input B with Transducer B leads to activation of either Reporter, whereas Input A results in no activation. This underscores that the selectivity of the system is encoded in the 4‐nt toehold overlap of the Input/Transducer recognition region. We note that for these experiments, Transducers and Reporters already contain the additional overhang sequences, which we will later use for binding into DNA condensates. The existence of these overhangs results in some unexpected hybridization with the Reporter toehold, which contributes to different reaction kinetics for different Reporters (Figure S2a–c). This effect can be avoided by hybridizing the Reporter overhangs with complementary strands (Figure S2d) and is therefore negligible for later in‐condensate DNA reaction cascade.

Most importantly, when both systems A and B are integrated in the same solution (overhang hybridized), interference and crosstalk appear (Figure 2e, see results for non‐hybridized overhang in Figure S2e). Upon adding any of the two Inputs (Input A or B), the corresponding Transducer is activated to release its Messenger into the whole solution, which then activates both Reporters (See Figure S3 for details of the reaction mechanism). It therefore becomes impossible to distinguish which Input is added. This minimalistic system highlights, by specific design, the general limitations of in‐solution DNA‐RNs, where sequence interference and crosstalk can occur between different DNA‐RNs. Parallel execution of Input‐specific DNA‐RNs would require redesign of the whole sequence set.

Now, let us compartmentalize the systems to overcome this limitation. We use micron‐sized core‐shell DNA condensates formed through liquid–liquid phase separation of long ssDNA, that, after fabrication, feature a liquid‐like interior with specific ssDNA barcodes to immobilize other DNA components while providing high intrinsic mobility [28, 29, 30, 31, 32, 33, 34, 35]. The shell is permeable and allows large macromolecules to penetrate [28, 36]. Using a recently developed co‐phase separation approach, it is possible to incorporate different ssDNA barcodes into the condensates at desired concentrations [34]. We designed and prepared two different DNA condensates (Figure S4). Condensate A contains a 1:1 ratio of barcodes m and p, whereas condensate B contains a 1:1 ratio of barcodes n and q. The local concentration of each barcode is around 200 µM [29].

**FIGURE 3:**
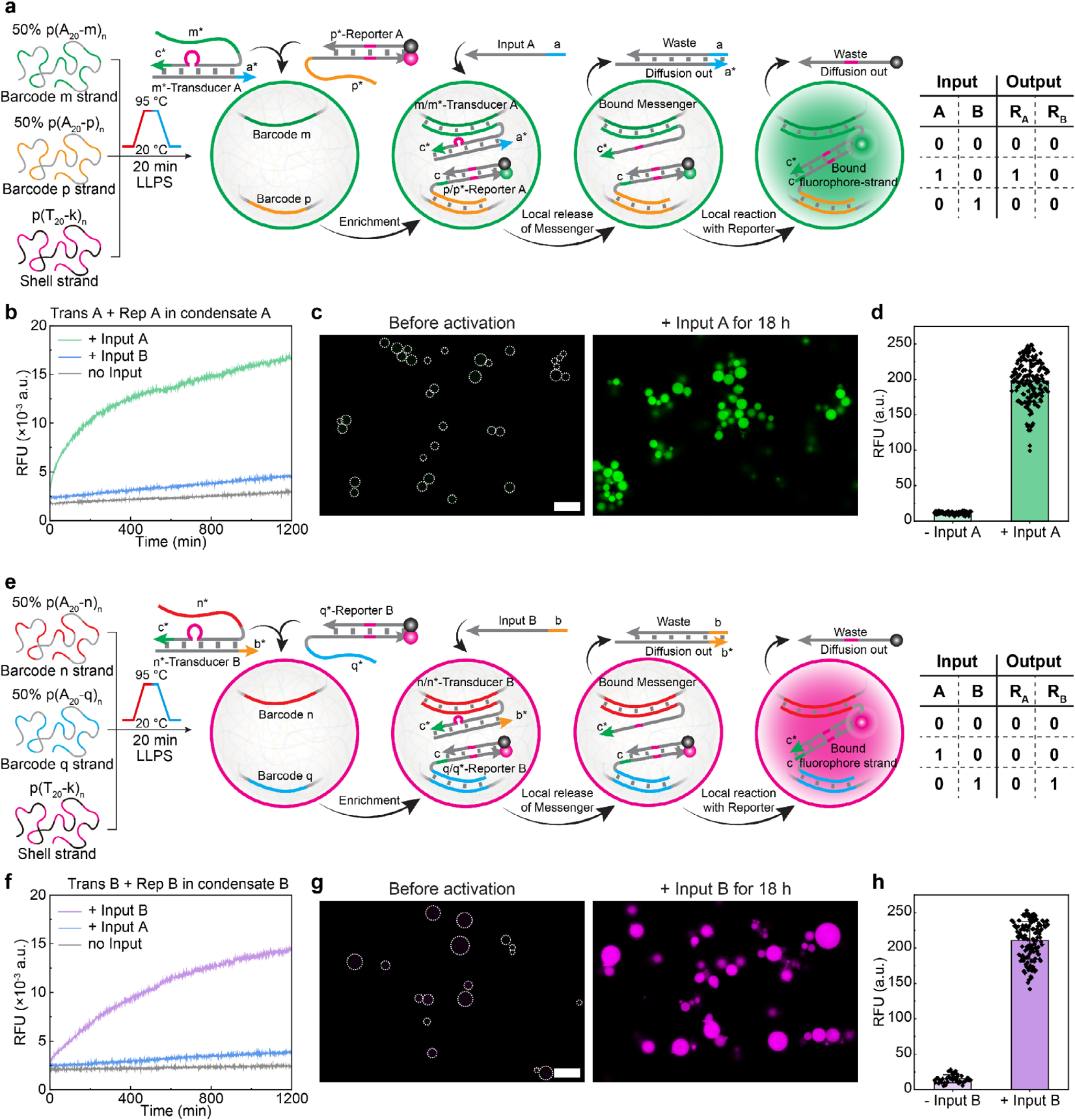
Liquid‐like DNA condensates support dynamic SDR reactions. (a) Scheme of a DNA condensate A containing m and p barcodes for encapsulating Transducer A and Reporter A with complementary overhang strands (m* and p*). The addition of Input A activates Transducer A to release a Messenger locally, which subsequently reacts with Reporter A in the same condensate. Corresponding truth table is provided. (b) Activation kinetics of Report A inside condensate A in response to different Inputs. (c) Representative CLSM images showing the activation of Reporter A in condensate A, triggered by Input A. (d) Fluorescence intensity inside DNA condensate A before and after adding Input A, as quantified from CLSM data in (c). (e) Scheme of a DNA condensate B containing n and q barcodes for encapsulating Transducer B and Reporter B with complementary overhang strands (n* and q*). The addition of Input B activates Transducer B to release a Messenger locally, which subsequently reacts with Reporter B in the same condensate. Corresponding truth table is provided. (f) Activation kinetics of Report B inside condensate B in response to different Inputs. (g) Representative CLSM images showing the activation of Reporter B in condensate B, triggered by Input B. (h) Fluorescence intensity inside DNA condensate B before and after adding Input B, as quantified from CLSM data in (g). *n* = 2 for plate reader experiments, *n* = 46–164 condensates measured from two independent samples for CLSM experiments. The curve represents the mean value. Error bars represent standard deviation. Scale bars are all 10 µm.

We then immobilized Transducer A and Reporter A into condensate A and Transducer B and Reporter B into condensate B via specific barcode binding (Figure 3a,e). The immobilization is done in a way that the important Messenger component remains tethered to the DNA condensate material and cannot escape the condensate by diffusion. Similarly, the dye‐labelled strand of the Reporter is attached to simplify imaging. We then evaluate the response of each condensate to Inputs A and B. FRAP experiments reveal high internal fluidity to execute DNA‐RNs based on the tethered strands (Figure S5). Both in‐condensate DNA‐RNs exhibit a positive response to their corresponding Inputs, resulting in activation of the embedded Reporter via a fluorescence signal (Figure 3b–d,f–h). As expected, the non‐complementary Input does not activate the Reporter. The minor increase in the intensity when adding wrong input may stem from a potential dynamic exchange between Transducer and Input due to the high local concentration of bound Transducer inside DNA condensates. These results demonstrate that DNA reactions can be efficiently carried out within the DNA condensates. The kinetics are comparable to those in solution, as efficient enrichment occurs through the permeable shells and on account of the dynamic condensate interior.

**FIGURE 4:**
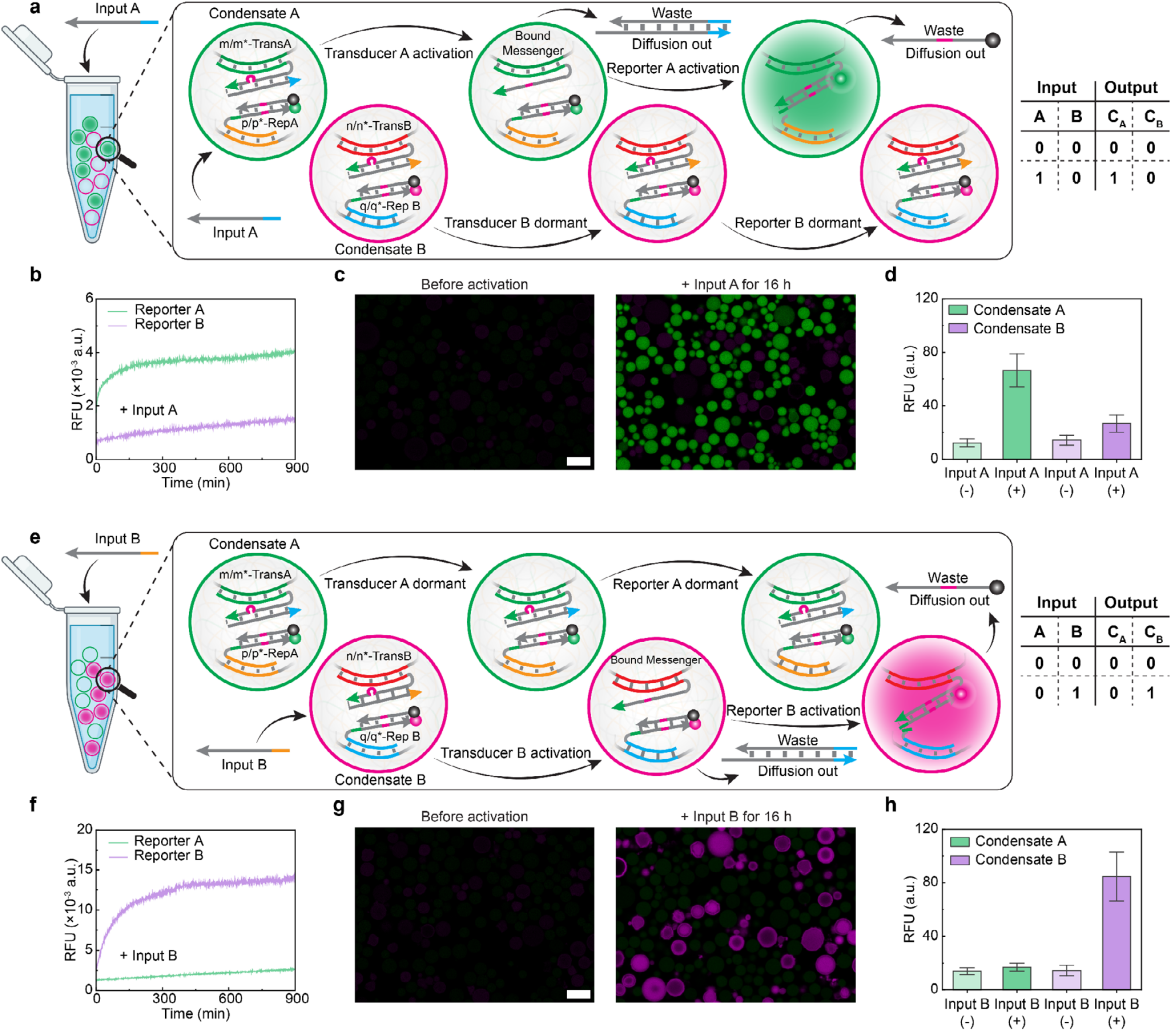
DNA condensates enable spatial confinement of DNA‐RNs. (a) Scheme of the parallel DNA reactions in two different DNA condensate populations, triggered by Input A, with the corresponding truth table. (b) The addition of Input A activates Reporter A embedded in condensate A with an increase in fluorescence signal, whereas Reporter B in condensate B remains dormant. (c) Representative CLSM images show that Input A specifically activates Reporter A in condensate population A, whereas Reporter B in condensate population B remains dormant. (d) Activation of Reporters A and B in both condensate populations in response to Input A, as quantified from CLSM data in (c). (e) Scheme of parallel DNA reactions in two different DNA condensate populations, triggered by Input B, with corresponding truth table. (f) The addition of Input B activates Reporter B embedded in condensate B with an increase in fluorescence signal, whereas Reporter A in condensate A remains dormant. (g) Representative CLSM images show that Input B specifically activates Reporter B in condensate population B, whereas Reporter A in condensate population A remains dormant. (h) Activation of Reporters A and B in both condensate populations in response to Input B, as quantified from CLSM data in (g). *C*_A_: Condensate A; *C*_B_: Condensate B. *n* = 2 for plate reader experiments, *n* > 200 condensates measured from two independent samples for CLSM experiments. The curve represents the mean value. Error bars represent standard deviation. Scale bars are all 10 µm. Created with BioRender.com.

Finally, we test the system behavior when both condensates are present with their embedded DNA‐RNs in the same solution. Strikingly, upon addition of Input A, only condensate A population processes the Input to activate the embedded Transducer A, which then triggers a local reaction with the internal Reporter A (Figure 4a,b). In contrast, Reporter B embedded in the condensate B population remains dormant. Complementary CLSM experiments further confirm these findings with the selective appearance of green fluorescence in condensate A while condensate B remains dark (Figure 4c,d). Conversely, addition of Input B triggers magenta fluorescence exclusively in condensates B, with condensates A remaining dark (Figure 4e–h). We note that there is a slight increase for the Reporters that are not supposed to be activated. This can be attributed to the existence of some free‐diffusing Transducers in the solution that were not fully purified away during centrifugation cleaning steps (see also Figure S6 for unpurified samples). However, this result is now profoundly different from the in‐solution experiment (Figure 2e), where either of the two Inputs activates both Reporters due to crosstalk on the Messenger level. These results unequivocally demonstrate that the compartmentalization in dynamic DNA condensates provides effective physical separation of interfering DNA‐RNs, enabling parallel and spatially confined DNA signal processing within the same solution. The localized activation of the Transducer and “release” of the tethered intermediate Messenger ensure that DNA reactions occur only within condensates capable to process the correct Input, and that any further signal processing remains confined within the compartment.

## 3 Conclusion

To summarize, we developed compartmentalized DNA‐RNs by using liquid‐like DNA condensates as hosts. Hybridization between condensate barcodes and DNA‐RN parts effectively governs localized DNA reactions within condensates and prevents the diffusive leakage of key components. This allows us to orthogonally operate DNA‐RNs having the same sequence design in different compartments without interference and crosstalk. Our strategy, therefore, eliminates the need for high orthogonality of DNA sequences in a large system. By introducing a Transducer module, different condensate‐specific Inputs can be converted to a unified Messenger for triggering downstream DNA‐RNs locally. This can be further exploited to couple with developed DNA‐RNs and facilitate their modular integration into a single system. More importantly, the permeable membrane of DNA condensates enables in‐condensate DNA‐RNs to exchange signals with the surrounding environment and between distinct condensates, as evidenced by Input uptake and Waste release (Figure 4a,e). This permeability can be further exploited to establish communications both between DNA condensates and their environment and across different condensate populations. In particular, we anticipate our research to stimulate development of DNA‐RNs with complex functions and DNA computing systems capable of operating a large number of DNA gates in the same solution [20]. Intriguingly, in the context of system chemistry, our methodology also demonstrates a simple design principle for biasing reaction pathways by exploiting the proximity effect in compartmentalized systems.

## Conflicts of Interest

The authors declare no conflicts of interest.

## Supporting information




**Supporting File 1**: anie72283‐sup‐0001‐SuppMat.pdf.

## Data Availability

The data that support the findings of this study are available from the corresponding author upon reasonable request.

## References

[1] L. Bar‐Peled and N. Kory , “Principles and Functions of Metabolic Compartmentalization,” Nature Metabolism 4 (2022): 1232–1244, 10.1038/s42255-022-00645-2. PMC10155461 36266543

[2] Y. Shin and C. P. Brangwynne , “Liquid Phase Condensation in Cell Physiology and Disease,” Science 357 (2017): eaaf4382, 10.1126/science.aaf4382. 28935776

[3] A. Zecchin , P. C. Stapor , J. Goveia , and P. Carmeliet , “Metabolic Pathway Compartmentalization: An Underappreciated Opportunity?,” Current Opinion in Biotechnology 34 (2015): 73–81, 10.1016/j.copbio.2014.11.022. 25499800

[4] C. P. Brangwynne , C. R. Eckmann , D. S. Courson , et al., “Germline P Granules are Liquid Droplets That Localize by Controlled Dissolution/Condensation,” Science 324 (2009): 1729–1732, 10.1126/science.1172046. 19460965

[5] A. A. Hyman , C. A. Weber , and F. Jülicher , “Liquid‐Liquid Phase Separation in Biology,” Annual Review of Cell and Developmental Biology 30 (2014): 39–58, 10.1146/annurev-cellbio-100913-013325. 25288112

[6] X. Li , X. Yang , J. Qi , and N. C. Seeman , “Antiparallel DNA Double Crossover Molecules as Components for Nanoconstruction,” Journal of the American Chemical Society 118 (1996): 6131–6140, 10.1021/ja960162o.

[7] N. C. Seeman , “DNA in a Material World,” Nature 421 (2003): 427–431, 10.1038/nature01406. 12540916

[8] W. B. Sherman and N. C. Seeman , “A Precisely Controlled DNA Biped Walking Device,” Nano Letters 4 (2004): 1203–1207, 10.1021/nl049527q.

[9] P. W. K. Rothemund , “Folding DNA to Create Nanoscale Shapes and Patterns,” Nature 440 (2006): 297–302, 10.1038/nature04586. 16541064

[10] N. C. Seeman and H. F. Sleiman , “DNA Nanotechnology,” Nature Reviews Materials 3 (2017): 1–23, 10.1038/natrevmats.2017.68.

[11] F. C. Simmel , B. Yurke , and H. R. Singh , “Principles and Applications of Nucleic Acid Strand Displacement Reactions,” Chemical Reviews 119 (2019): 6326–6369, 10.1021/acs.chemrev.8b00580. 30714375

[12] G. Seelig , D. Soloveichik , D. Y. Zhang , and E. Winfree , “Enzyme‐Free Nucleic Acid Logic Circuits,” Science 314 (2006): 1585–1588, 10.1126/science.1132493. 17158324

[13] L. Qian and E. Winfree , “Scaling up Digital Circuit Computation With DNA Strand Displacement Cascades,” Science 332 (2011): 1196–1201, 10.1126/science.1200520. 21636773

[14] D. A. Khodakov , A. S. Khodakova , A. Linacre , and A. V. Ellis , “Toehold‐Mediated Nonenzymatic DNA Strand Displacement as a Platform for DNA Genotyping,” Journal of the American Chemical Society 135 (2013): 5612–5619, 10.1021/ja310991r. 23548100

[15] M. You , G. Zhu , T. Chen , M. J. Donovan , and W. Tan , “Programmable and Multiparameter DNA‐Based Logic Platform for Cancer Recognition and Targeted Therapy,” Journal of the American Chemical Society 137 (2015): 667–674, 10.1021/ja509263k. 25361164 PMC4308741

[16] C. Sharma , A. Samanta , R. S. Schmidt , and A. Walther , “DNA‐Based Signaling Networks for Transient Colloidal Co‐Assemblies,” Journal of the American Chemical Society 145 (2023): 17819–17830, 10.1021/jacs.3c04807. 37543962

[17] S. Groeer , K. Schumann , S. Loescher , and A. Walther , “Molecular Communication Relays for Dynamic Cross‐Regulation of Self‐Sorting Fibrillar Self‐Assemblies,” Science Advances 7 (2021): eabj5827, 10.1126/sciadv.abj5827. 34818037 PMC8612681

[18] J. Deng and A. Walther , “Autonomous DNA Nanostructures Instructed by Hierarchically Concatenated Chemical Reaction Networks,” Nature Communications 12 (2021): 5132, 10.1038/s41467-021-25450-5. PMC8390752 34446724

[19] S. Gentile , E. Del Grosso , P. E. Pungchai , E. Franco , L. J. Prins , and F. Ricci , “Spontaneous Reorganization of DNA‐Based Polymers in Higher Ordered Structures Fueled by RNA,” Journal of the American Chemical Society 143 (2021): 20296–20301, 10.1021/jacs.1c09503. 34843256 PMC8662731

[20] S. D. Lemaire , D. Turek , D. Landsman , M. Colotte , and T. F. A. de Greef , “Challenges and Opportunities in DNA Computing and Data Storage,” Nature Nanotechnology 20 (2025): 710–714, 10.1038/s41565-025-01937-w. 40494931

[21] H. Lv , N. Xie , M. Li , et al., “DNA‐based Programmable Gate Arrays for General‐Purpose DNA Computing,” Nature 622 (2023): 292–300, 10.1038/s41586-023-06484-9. 37704731

[22] G. Chatterjee , N. Dalchau , R. A. Muscat , A. Phillips , and G. Seelig , “A Spatially Localized Architecture for Fast and Modular DNA Computing,” Nature Nanotechnology 12 (2017): 920–927, 10.1038/nnano.2017.127. 28737747

[23] H. Bui , S. Shah , R. Mokhtar , T. Song , S. Garg , and J. Reif , “Localized DNA Hybridization Chain Reactions on DNA Origami,” ACS Nano 12 (2018): 1146–1155, 10.1021/acsnano.7b06699. 29357217

[24] S. Do , C. Lee , T. Lee , D.‐N. Kim , and Y. Shin , “Engineering DNA‐Based Synthetic Condensates With Programmable Material Properties, Compositions, and Functionalities,” Science Advances 8 (2022): eabj1771, 10.1126/sciadv.abj1771. 36240277 PMC9565806

[25] J. Gong , N. Tsumura , Y. Sato , and M. Takinoue , “Computational DNA Droplets Recognizing miRNA Sequence Inputs Based on Liquid–Liquid Phase Separation,” Advanced Functional Materials 32 (2022): 2202322, 10.1002/adfm.202202322.

[26] Q.‐H. Zhao , F.‐H. Cao , Z.‐H. Luo , W. T. S. Huck , and N.‐N. Deng , “Photoswitchable Molecular Communication Between Programmable DNA‐Based Artificial Membraneless Organelles,” Angewandte Chemie International Edition 61 (2022): e202117500, 10.1002/anie.202117500. 35090078

[27] E. Kengmana , E. Ornelas‐Gatdula , K.‐L. Chen , and R. Schulman , “Spatial Control Over Reactions via Localized Transcription Within Membraneless DNA Nanostar Droplets,” Journal of the American Chemical Society 146 (2024): 32942–32952, 10.1021/jacs.4c07274. 39565729 PMC11701973

[28] A. Samanta , V. Sabatino , T. R. Ward , and A. Walther , “Functional and Morphological Adaptation in DNA Protocells via Signal Processing Prompted by Artificial Metalloenzymes,” Nature Nanotechnology 15 (2020): 914–921, 10.1038/s41565-020-0761-y. PMC7610402 32895521

[29] W. Chen , B. Dúzs , P. G. Argudo , et al., “Ballistic Diffusion Fronts in Biomolecular Condensates,” Nature Nanotechnology 20 (2025): 1062–1070, 10.1038/s41565-025-01941-0. PMC12373509 40481268

[30] W. Liu , C. Lupfer , A. Samanta , A. Sarkar , and A. Walther , “Switchable Hydrophobic Pockets in DNA Protocells Enhance Chemical Conversion,” Journal of the American Chemical Society 145 (2023): 7090–7094, 10.1021/jacs.3c00997. 36971596

[31] R. Merindol , S. Loescher , A. Samanta , and A. Walther , “Pathway‐Controlled Formation of Mesostructured all‐DNA Colloids and Superstructures,” Nature Nanotechnology 13 (2018): 730–738, 10.1038/s41565-018-0168-1. PMC6082344 29941888

[32] W. Liu , A. Samanta , J. Deng , C. O. Akintayo , and A. Walther , “Mechanistic Insights Into the Phase Separation Behavior and Pathway‐Directed Information Exchange in All‐DNA Droplets,” Angewandte Chemie International Edition 61 (2022): e202208951, 10.1002/anie.202208951. 36112754 PMC9828218

[33] A. Samanta , M. Hörner , W. Liu , W. Weber , and A. Walther , “Signal‐Processing and Adaptive Prototissue Formation in Metabolic DNA Protocells,” Nature Communications 13 (2022): 3968, 10.1038/s41467-022-31632-6. PMC9270428 35803944

[34] W. Chen , S. Song , A. Samanta , et al., “Growing Functional Artificial Cytoskeletons in the Viscoelastic Confinement of DNA Synthetic Cells,” Nature Chemical Engineering 2 (2025): 627–639, 10.1038/s44286-025-00289-5. PMC12545198 41142504

[35] W. Chen , J. Fritzen , and A. Walther , “Phase Separation of Nucleic Acids: Mechanisms, Properties, and Applications,” Angewandte Chemie International Edition 65 (2026): e23943, 10.1002/anie.202523943. 41636358 PMC12970503

[36] M. Xie , W. Chen , M. Vonk‐de Roy , and A. Walther , “Constructing Synthetic Nuclear Architectures via Transcriptional Condensates in a DNA Protonucleus,” Nature Communications 16 (2025): 8254, 10.1038/s41467-025-63445-8. PMC12423304 40931032

