# Supplementary material for: DNA Condensates Enable Crosstalk‐Free Operation of Identical DNA Computing Cascades
Source: Angew Chem Int Ed Engl. 2026 Apr 20;65(22):e5954994. doi: 10.1002/anie.5954994 (PMC13206538; doi:10.1002/anie.5954994)
Supplement: Supplementary file 1 — Supporting File 1: anie72283‐sup‐0001‐SuppMat.pdf. [file ANIE-65-e5954994-s001.pdf]

Supporting Information

DNA Condensates Enable Crosstalk-Free Operation of Identical DNA Computing Cascade

Weixiang Chen*,[a,b] Rahmetullah Demirci,[a] Miao Xie,[a,b] and Andreas Walther*[a,b]

[a] W. Chen, R. Demirci, Dr. M. Xie, Prof. Dr. A. Walther
Life-Like Materials and Systems, Department of Chemistry
University of Mainz
Duesbergweg 10–14, 55128 Mainz (Germany)
E-mail: weixiang.chen@uni-mainz.de; andreas.walther@uni-mainz.de

[b] W. Chen, Dr. M. Xie, Prof. Dr. A. Walther
Max Planck Institute for Polymer Research
Ackermannweg 10, 55128 Mainz (Germany)

Table of Contents

1. Materials 3

2. Instruments.. 3

3. Oligonucleotide sequences 3

4. Experimental methods.. 4

5. Supplementary Figures 7

1. Materials

Single-stranded DNA (ssDNA) were purchased from Biomers and Integrated DNA Technologies (IDT). T4 DNA Ligase (2 U/μL), Exonuclease I (40 U/μL), Exonuclease III (200 U/μL), and Φ_{29} polymerase (10 U/μL) were purchased from LGC Genomics. Thermal stable inorganic pyrophosphatase (2 U/μL) and nuclease-free water were bought from New England BioLabs (NEB). Deoxynucleotide triphosphate (dATP, dTTP, dGTP and dCTP) (100 mM) were purchased from Jena Bioscience. Hexadecane, sodium chloride and magnesium chloride, and Magnesium acetate solution (1M in water) were purchased (as bioreagent grade if available) from Sigma-Aldrich. RNase-free TE buffer (Invitrogen, 10 mM Tris and 1 mM EDTA, pH 8.0, 500 mL) were purchased from ThermoFisher Scientific. Phosphate-buffered saline (PBS) were purchased from CARL ROTH. 384 well high-content imaging glass bottom microplates for microscopy and 384 well microplates for plate reader were purchased from Corning.

2. Instruments

All thermal annealing and heating ramps were performed on a TPersonal Thermocycler (Analytik Jena). Incubation was carried out on an Eppendorf ThermoMixer C. DNA concentrations were determined by a DS-11 Spectrophotometer (DeNovix). Fluorescence measurements were performed by a plate reader (Tecan). Confocal laser scanning microscopy (CLSM) was performed on a Leica Stellaris 5.

3. Oligonucleotide sequences

Oligomers for condensates, labels, and DNA reaction networks, with their names, sequences, purification methods, modifications, and suppliers.

	Name	Sequence (5’ → 3’)	Purification	Modification	Supplier
Template and RCA	Tp(A_{20}-m)	/Phosphate/ATC TAT CCT AAT TTT TTT TTT TTT TTT TTT TGA ACC CGT AT	HPLC	5’-Phosphorylation	Biomers
	Tp(A_{20}-p)	/Phosphate/ATA GTG AGT CGT ATT ATT TTT TTT TTT TTT TTT TTT ATC CCT	HPLC	5’-Phosphorylation	Biomers
	Tp(A_{20}-n)	/Phosphate/TCG TTC AGA TGT TTT TTT TTT TTT TTT TTT TTG GGG TCC TTA C	HPLC	5’-Phosphorylation	Biomers
	Tp(T_{20}-q)	/Phosphate/TAT AAG TGC CAT TTT TTT TTT TTT TTT TTT TTC TTC CGA CAG C	HPLC	5’-Phosphorylation	Biomers
	Tp(T_{20}-k)	/Phosphate/ATC CTC TAA AAT CAA AAA AAA AAA AAA AAA AAG TAA AAC CAC ACG	HPLC	5’-Phosphorylation	Biomers
	ligation-m	TTA GGA TAG ATA TAC GGG TTC	HPLC	None	Biomers
	ligation-p	TAA TAC GAC TCA CTA TAG GGA T	HPLC	None	Biomers
	ligation-n	CAT CTG AAC GAG TAA GGA CCC CA	HPLC	None	Biomers
	ligation-q	TGG CAC TTA TAG CTG TCG GAA GA	HPLC	None	Biomers
	ligation-k	TTT TAG AGG ATC GTG TGG TTT T	HPLC	None	Biomers
	primer-m	TTA GGA TAG ATA TAC GGG T*T*C	Desalting	Phosphorothioated twice (*)	IDT
	primer-p	TAA TAC GAC TCA CTA TAG GG*A*T	Desalting	Phosphorothioated twice (*)	IDT
	primer-n	CAT CTG AAC GAG TAA GGA CCC*C*A	Desalting	Phosphorothioated twice (*)	IDT

	primer-q	TGG CAC TTA TAG CTG TCG GAA*G*A	Desalting	Phosphorothioated twice (*)	IDT
	primer-k	TTT TAG AGG ATC GTG TGG TT*T*T	Desalting	Phosphorothioated twice (*)	IDT
Barcode label	Atto488-m*	/ATTO488/TGA ACC CGT ATA TCT ATC CTA A	HPLC	5’-Atto488	Biomers
	Atto565-p*	/ATTO565/ATC CCT ATA GTG AGT CGT ATT A	HPLC	5’-Atto565	Biomers
	Atto565-n*	/ATTO565/-TGG GGT CCT TAC T	HPLC	5’-Atto565	Biomers
	Atto425-q*	/ATTO425/ TCT TCC GAC AGC TAT AAG TGC CA	HPLC	5’-Atto425	Biomers
	Atto647-k*	/ATTO647N/AAA ACC ACA CGA TCC TCT AAA A	HPLC	5’-Atto647N	Biomers
Inputs, Transducers, Reporters	A1 (Transducer A)	TAT AGC CAT AAA TGA CAA CCG TGC AGT	HPLC	None	Biomers
	m*-TTT-A2 (Transducer A’)	GAA CCC GTA TAT CTA TCC TAA TTT CAC GGT TGT CAT TTC GAT GGA TGG CTA TA	HPLC	None	Biomers
	p*-A3-FAM (Reporter A)	ATC CCT ATA GTG AGT CGT ATT ATT TTA TAG CCA TCC ATC GAA ATG ACAA/6-FAM/	HPLC	3’-6-FAM	Biomers
	A4 (Reporter A’)	/BBQ535/TTG TCA TTT CGA TGG ATG G	HPLC	5’-BBQ535	Biomers
	A5 (Input A)	ACT GCA CGG TTG TCA TTT ATG	HPLC	None	Biomers
	B1 (Transducer B)	TAT AGC CAT AAA TGA CAA CCG TGG GTT	HPLC	None	Biomers
	n*-TTT-B2 (Transducer B’)	TGG GGT CCT TAC TCG TTC AGA TGT TTC ACG GTT GTC ATT TCG ATG GAT GGC TAT A	HPLC	None	Biomers
	q*-B3-Cy5 (Reporter B)	CTT CCG ACA GCT ATA AGT GCC ATT TTA TAG CCA TCC ATC GAA ATG ACAA/Cy5/	HPLC	3’-Cy5	Biomers
	B4 (Reporter B’)	/BBQ650/TTG TCA TTT CGA TGG ATG G	HPLC	5’-BBQ650	Biomers
	B5 (Input B)	AAC CCA CGG TTG TCA TTT ATG	HPLC	None	Biomers
	p	TAA TAC GAC TCA CTA TAG GGA T	HPLC	None	Biomers
	q	TGG CAC TTA TAG CTG TCG GAA GA	HPLC	None	Biomers

4. Experimental methods

Synthesis of circular ssDNA templates and long ssDNA polymers

Synthesis of circular DNA template and its corresponding ssDNA polymer is adapted from our previous reports. The linear ssDNA template and the corresponding ligation strand were firstly mixed at concentration of 1 μM in 100

μL TE buffer containing 100 mM NaCl. The solution was heated to 85 °C (3 °C/s) for 5 min before cooling to 25 °C (0.01 °C/s) for complete hybridization. Afterwards, 20 μL of 10× Ligase buffer (500 mM Tris-HCl, 100 mM $MgCl_2$, 50 mM dithiothreitol and 10 mM ATP (Lucigen)), 70 μL of nuclease-free water and 10 μL of T4 DNA Ligase (2 U/μL (Lucigen)) were introduced into the reaction mixture and gently mixed before leaving at room temperature for 3 h. The reaction mixture was then heated to 70 °C for 20 min to deactivate the enzyme. Then, 10 μL of Exonuclease I (40 U/μL (Lucigen)) and 10 μL of Exonuclease III (200 U/μL (Lucigen)) were added into the reaction mixture to react overnight at 37 °C for degradation of the ligation strands and any non-circularized templates in solution. Afterwards, the reaction mixture was heated to 80 °C for 40 min to deactivate the enzymes. To obtain the final circular ssDNA templates, the reaction mixture was washed by adding 400 μL TE buffer and filtrated using Amicon Ultra-centrifugal filters with a 10 kDa cut-off (Merck Millipore) for three times. The concentrations of the collected circular ssDNA templates were measured by the DS-11 Spectrophotometer (DeNovix), and the templates were stored in TE buffer at -20 °C.

For synthesis of long ssDNA polymers, we used rolling circle amplification (RCA). 5 μL of circular template (1 μM in TE buffer) and 1 μL of exonuclease resistant primer (10 μM in TE buffer) were mixed with 76 μL nuclease-free water, 10 μL of commercial 10× polymerase buffer (500 mM Tris-HCl, 100 mM $(NH_4)_2SO_4$, 40 mM dithiothreitol, 100 mM $MgCl_2$ (Lucigen)), 2 μL of Φ_{29} DNA polymerase (10 U/μL (Lucigen)), 1 μL of thermal stable inorganic pyrophosphatase (2 U/μL (NEB)) and 5 μL of adjusted deoxyribose nucleoside 5'-triphosphate mix (100 mM, the mix contains pure dATP, dTTP, dCTP, and dGTP solutions mixed in corresponding proportions of the exact composition of the desired ssDNA polymer repeating units (Jena Bioscience)). The reaction mixture was kept at 30 °C for 65 h before thermal cleavage at 95 °C for 15 min to shorten the ultrahigh molecular weight ssDNA. The final products were purified by rinsing with 400 μL TE buffer and filtration in Amicon Ultra-centrifugal filters with 30 kDa cut-off (Merck Millipore) for three times. The concentrations of the collected final ssDNA polymers were measured using the DS-11 Spectrophotometer (DeNovix), and the DNA polymers (cleaved at 95 °C for 15 min) were stored in TE buffer at -20 °C.

Preparation of DNA condensates via *co*-phase separation

To prepare DNA condensate containing 50% m barcode and 50% p barcode, 0.222 g/L of $p(A_{20}\text{-}m)_n$, 0.222 g/L of $p(A_{20}\text{-}p)_n$, and 0.111 g/L $p(T_{20}\text{-}k)_n$ were mixed in TE buffer without any salt at a final volume of 18 μL. The solution mixture was then heated at 95 °C for 15 min (in addition to the first thermal cleavage during ssDNA synthesis mentioned above) for thermal cleavage to further reduce the chain length of the ssDNA polymers. Afterwards, 2 μL of TE buffer containing 500 mM $MgCl_2$ was introduced into the reaction mixture. The solution containing finally 0.2 g/L $p(A_{20}\text{-}m)_n$, 0.2 g/L $p(A_{20}\text{-}p)_n$, and 0.1 g/L $p(T_{20}\text{-}k)_n$ with 50 mM $MgCl_2$ was heated to 95 °C for 20 min (to induce $p(A_{20}\text{-}m)_n$ and $p(A_{20}\text{-}p)_n$ phase separation) and subsequently cooled down to room temperature, during which the $p(T_{20}\text{-}k)_n$ localized as shell on the periphery of the $p(A_{20}\text{-}m)_n$ and $p(A_{20}\text{-}p)_n$ core by A_{20}/T_{20} hybridization. The heating and cooling rates were both 3 °C/s. Finally, the 20 μL solution containing the core-shell DNA condensates was diluted 2 times by adding 20 μL PBS buffer. The obtained 40 μL DNA condensate solution (as 2 times diluted) has 0.1 g/L $p(A_{20}\text{-}m)_n$, 0.1 g/L $p(A_{20}\text{-}p)_n$, and 0.05 g/L $p(T_{20}\text{-}k)_n$, corresponding to ca. 8 μM m barcode, 8 μM p barcode, and 4 μM k barcode, respectively, in total solution. The solution was then stored in a fridge at 4 °C for 1 week for equilibration. Before usage, the DNA condensate solution was always slightly vortexed to redisperse the DNA SCs homogenously in the solution. For preparation of DNA condensates containing 50% n barcode and 50% q barcode, the $p(A_{20}\text{-}m)_n$ and $p(A_{20}\text{-}p)_n$ were replaced by $p(A_{20}\text{-}n)_n$ and $p(A_{20}\text{-}q)_n$. The successful integration of the desired barcodes in the DNA condensates was then confirmed by CLSM (Supplementary Figure 4).

Fluorescence recovery after photobleaching

FRAP experiments were performed independently on various labeled barcodes (m, p, n, q) of different DNA condensates to probe the internal dynamics of DNA condensates. Condensate barcodes are labeled by 2× stoichiometric amount of DNA strands tagged with different fluorophores (Atto488-m*, Atto565-p*, Atto565-n*, Atto425-q*). Condensates were firstly imaged with low laser intensity 5 times (1 s/frame) as pre-bleaching images, before 5 times bleaching by 100% laser intensity in a circular ROI with a diameter of 1.5 μm in the center of condensates. Then, 60 frames of post-bleaching images were recorded at a rate of 1 s/frame. For quantification, we measured intensities within the bleached circular ROI (I_{ROI}), and intensities in a circular ROI away from bleached

region within the condensates (I_{ref}), in pre- and post-bleaching images. We applied double normalization by $I_{\mathrm{Norm}}(t) = \frac{I_{\mathrm{ROI}}(t)}{I_{\mathrm{ROI}}(t_0)} \times \frac{I_{\mathrm{ref}}(t_0)}{I_{\mathrm{ref}}(t)}$, where $I(t_0)$ represents the intensity measured in the first image before bleaching and $I(t)$ is the intensity measured over time.

Preparation of DNA Transducer and Reporter

Double-stranded DNA duplexes such as Transducer and Reporter are prepared by heating a solution containing 20 µM of each of the needed strands in TE buffer containing 15 mM $MgCl_2$ to 85 °C for 5 min and then cooling down to 20 °C (0.1 °C/min). The formed DNA duplex solutions were stored at -20 °C for further experiments.

DNA reaction networks in solution and in DNA condensates

Isolated DNA reaction networks in solution were prepared by mixing 400 nM Transducer A (or B), 400 nM Reporter A (or B) in 20 µL PBS buffer containing 15 mM $MgAc_2$ in well plate. After 1h incubation, the reaction networks were triggered by the addition of 800 nM (2× stoichiometric) Input A (or B) and monitored by plate reader. Depending on specific experiments, 600 nM p strands and 600 nM q strands can be added for hybridizing p* overhang of Reporter A, and q* overhang of Reporter B, respectively.

Combined DNA reaction networks in solution were prepared by mixing 400 nM Transducer A, 400 nM Transducer B, 400 nM Reporter A, 400 nM Reporter B in 20 µL PBS buffer containing 15 mM $MgAc_2$ in well plate. In addition, 600 nM p strands and 600 nM q strands were added for hybridizing p* overhang of Reporter A, and q* overhang of Reporter B, respectively. After 2h incubation, the reaction networks were triggered by the addition of 800 nM (2× stoichiometric) Input A (or B) and monitored by plate reader. Note that the addition of ssDNA p and q are important for preventing Reporter overhangs from forming secondary structures and blocking the toehold. The absence of p and q ssDNA in such reaction systems leads to an unbalanced reaction (see Supplementary Figure 2).

Isolated DNA reaction networks in DNA condensates were prepared by mixing 1 µL of condensate A (or B) stock solution (corresponding to 400 nM of each barcode in final solution) with 400 nM Transducer A (or B), 400 nM Reporter A (or B) in 20 µL PBS buffer containing 15 mM $MgAc_2$ in well plate. The samples were incubated in well plate for 6h before the addition of 800 nM (2× stoichiometric) Input A (or B) for triggering the system and monitored by plate reader and CLSM.

Combined DNA reaction networks of both systems in DNA condensates were prepared by mixing 2 µL of condensate A stock solution and 2 µL of condensate B stock solution with 800 nM Transducer A, 800 nM Transducer B, 800 nM Reporter A, and 800 nM Reporter B in 20 µL PBS buffer containing 15 mM $MgAc_2$ in well plate. The samples were incubated in well plate for 6h, before 4 times washing process by adding 20 µL PBS buffer containing 15 mM $MgAc_2$, centrifugation, and removal of 20 µL supernatant to remove excess DNA duplexes and potentially free DNA polymers in solution. Afterwards, the system was triggered by an addition of 800 nM (1× stoichiometric) Input A (or B) and monitored by plate reader and CLSM. Please note that the washing process is essential to remove all excess strands to avoid interference and crosstalk and ensure parallel DNA reactions in different DNA condensates (Supplementary Figure 6).

Gel electrophoresis for reaction characterization

DNA reaction system A in solution was prepared by mixing 400 nM Transducer A, 400 nM Reporter A, 800 nM Input A in 20 µL PBS buffer containing 15 mM $MgAc_2$ in a PCR tube. After 20 h reaction, this sample was analyzed by gel electrophoresis with 3 wt% agarose gel under 5 V/cm for 150 min in TAE buffer containing 40 mM Tris-HCl, 20 mM acetic acid and 1 mM EDTA. In addition to this sample, 800 nM Input A, 400 nM Transducer A, and 400 nM Reporter A were added in different lanes as references. Pre-staining by SYBR gold was used.

Statistical analysis

All data points featuring statistical analysis are presented as mean or mean ± standard deviation. The sample size (n) for each statistical analysis is reported in the relevant figure legend. Statistical analysis was carried out using Origin 2023 and Microsoft Excel.

5. Supplementary Figures

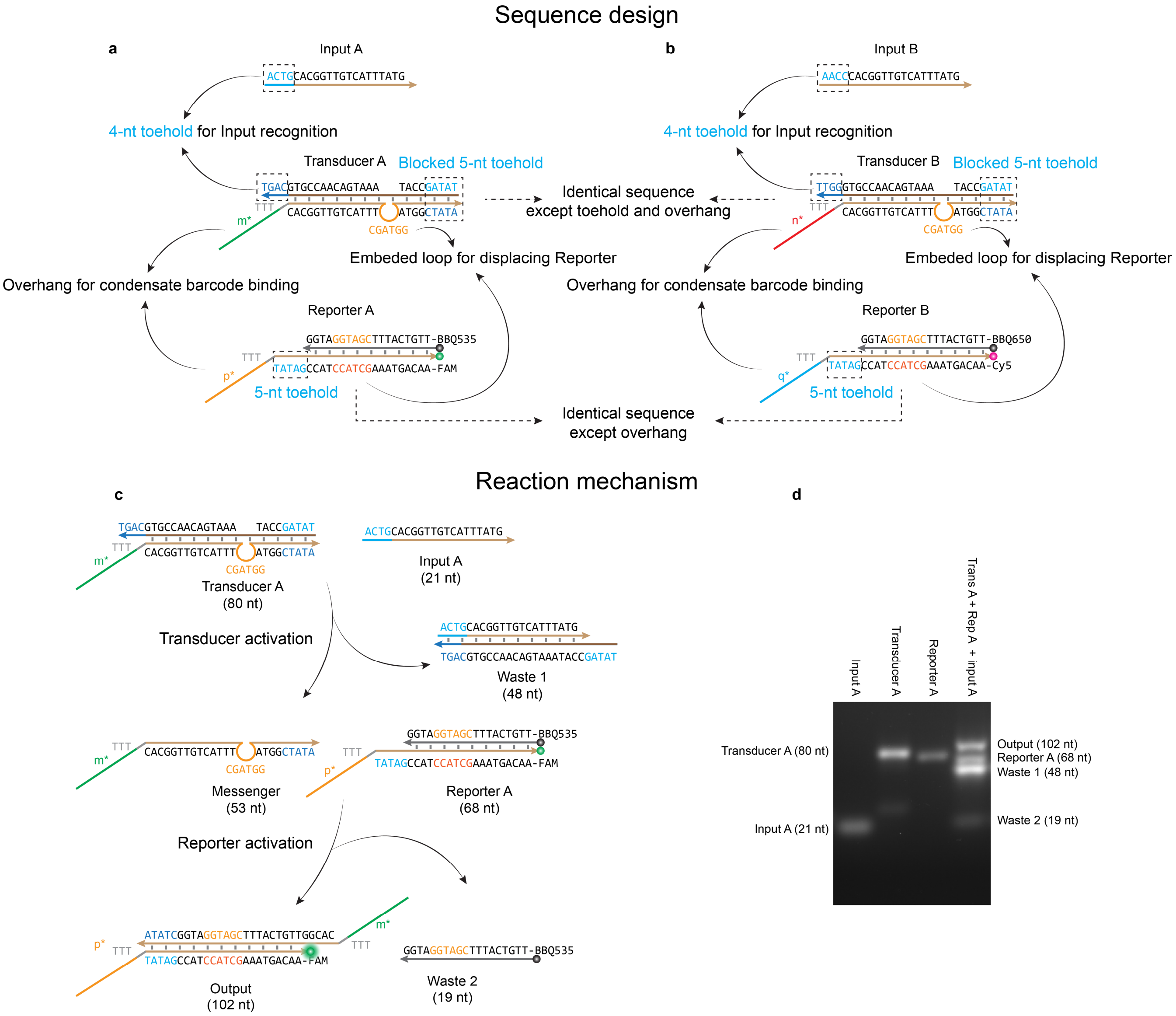

Supplementary Figure 1. Sequen design and reaction mechanism for Transducer and Reporter. (a) Sequence design for Transducer A and Reporter A. (b) Sequence design for Transducer B and Reporter B. (c) Reaction mechanism for Transducer A and Reporter A in isolated solution. (d) Gel electrophoresis analysis of the reaction for system A triggered by Input A. The toehold domains are highlighted in dashed boxes.

Note that there are three main principles for designing the Input-Transducer-Reporter reaction cascade inside DNA condensates:

1. **Overhang complementary to DNA condensate barcode:** To compartmentalize DNA reaction cascade and to keep the essential strands in the DNA condensates during the reaction cascade, the overhang domain (complementary to the DNA condensate barcode) of each DNA complex (such as Transducer and Reporter) should be allocated to the key RN strands for preventing their diffusion out of condensates and for enabling local reaction after activation. Taking Transducer as an example, the overhang is assigned to the unified Messenger so that it does not diffuse out upon activation and stays in the condensates to react with the

Reporter locally. If the overhang is assigned to the counterpart of the Messenger, it will lead to the diffusion of Messenger out of DNA condensates and trigger a global reaction among all DNA condensates. For the Reporter, the overhang is assigned to the fluorophore strand for direct visualization of the reaction inside DNA condensates.

2. **Input-Transducer reaction design and non-bonded loop domain of the Messenger:** Input should not directly react with Reporter. The reaction cascade to the Reporter is mediated through the Transducer for scaling Input diversity. This leads to a necessary non-bonded loop structure in the Messenger of Transducer, which should not be complementary to the Input, but to the Reporter. This design avoids significant sequence overlaps between Input and Reporter but still enables the release of the Messenger when Input binds to the Transducer. The non-bonded loop region further adds to the complementary domains to the Reporter and therefore facilitates the subsequent reaction with Reporter. It should be noted that this non-bonded loop domain must **not** be complementary to the toehold part (but rather the hybridized part) of the Reporter to avoid their direct interaction before Input addition. The release of the Messenger could be more efficient with longer toehold domains between Input and Transducer. In our paper, we used 4-nt toehold for simplicity and proof of concept, which could potentially be further extended to 6-nt or 8-nt toehold, etc.
3. **Transducer-Reporter reaction design:** The toehold domain on the Reporter should not react with the unreleased Messenger of Transducer. This is achieved by blocking the toehold-complementary domains of the Messenger in hybridized state. This domain is only available once Input is added to trigger the release of Messenger.

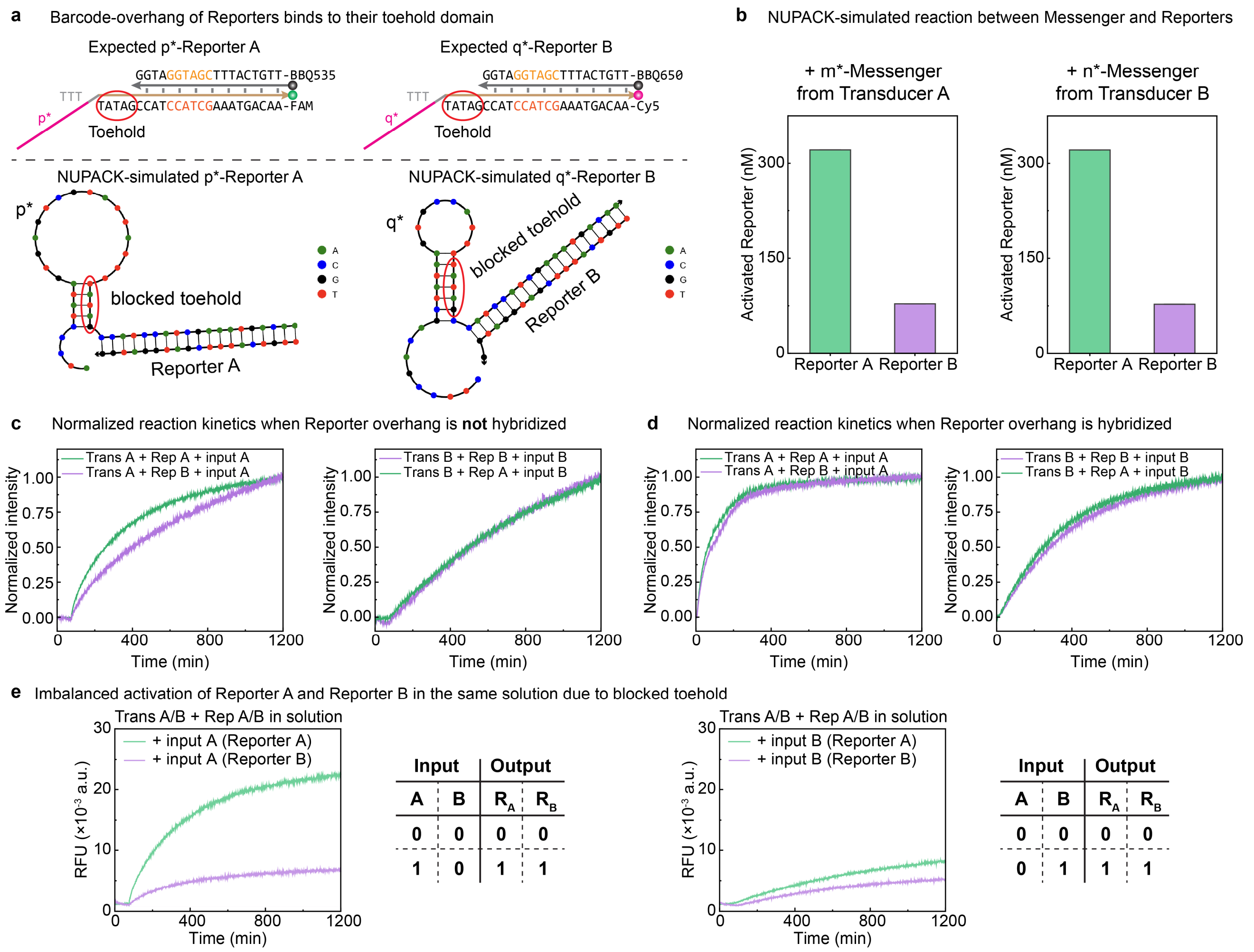

Supplementary Figure 2. Barcode-overhang of Reporters binds to their toehold domain and influences the reaction kinetics. (a) NUPACK simulation reveals that the barcode-overhang of Reporters binds to their toehold domains for reacting with Messengers. Reporter A's toehold is blocked with 5 bp binding whereas Reporter B's toehold is blocked with 7 bp binding. (b) NUPACK simulations reveal that the Messenger preferentially reacts with Reporter A, owing to weaker blocking of the toehold (5 bp < 7 bp). (c) Normalized reaction kinetics for in-solution DNA reaction cascade with the right input. When Reporter overhang is not hybridized, Reporter A and Reporter B show different reaction kinetics. (d) Normalized reaction kinetics for in-solution DNA reaction cascade with the right input. When Reporter overhang is hybridized, Reporter A and Reporter B show similar reaction kinetics. (e) In case of Transducers A, B and Reporters A, B in the same solution, any of both Inputs A and B reacts with its corresponding Transducer to release a Messenger in the whole solution, which activates both Reporters A and B. However, Reporter A has a faster reaction kinetics than Reporter B due to its weaker blocking of the toehold. **Such reaction kinetics difference can be addressed by binding the barcodes with complementary strands, so that both Reporters have the same free toeholds to react with the Messenger.** *n* = 2 for (c)–(e). The curve represents the mean value.

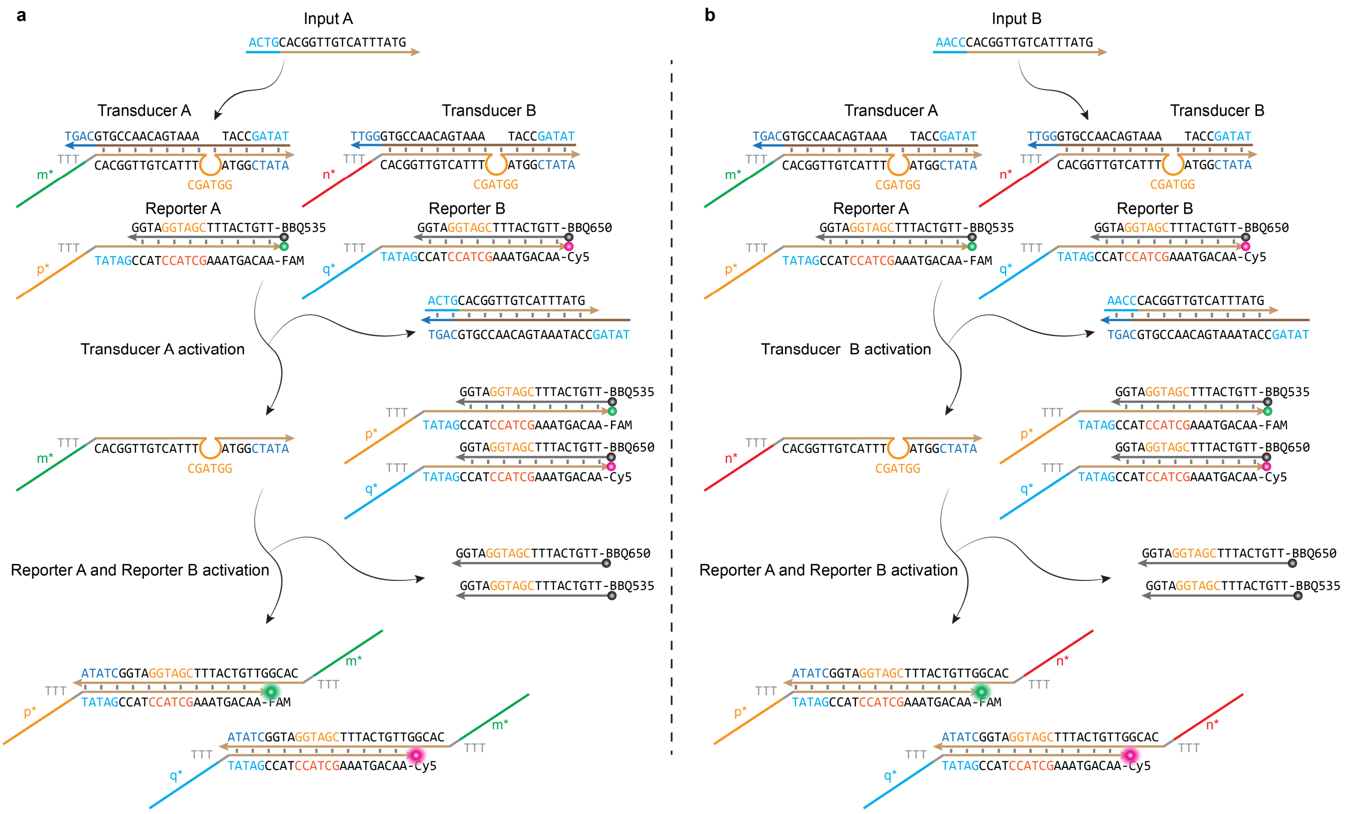

Supplementary Figure 3. Reaction mechanism when executing both system A and system B in the same solution. (a) Scheme illustrating that addition of Input A will result in the activation of both system A and system B. (b) Scheme illustrating that addition of Input B will result in the activation of both system A and system B.

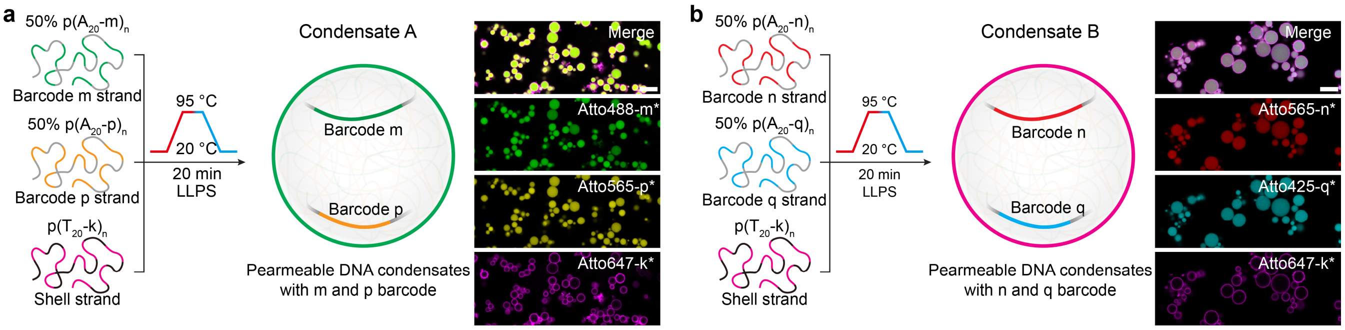

Supplementary Figure 4. Preparation of DNA condensates containing different barcodes. (a) Scheme and CLSM images for the DNA condensate A with m and p barcodes, labeled by Atto488-m* and Atto565-p*, respectively. (b) Scheme and CLSM images for the DNA condensate B with n and q barcodes, labeled by Atto565-n* and Atto425-q*, respectively.

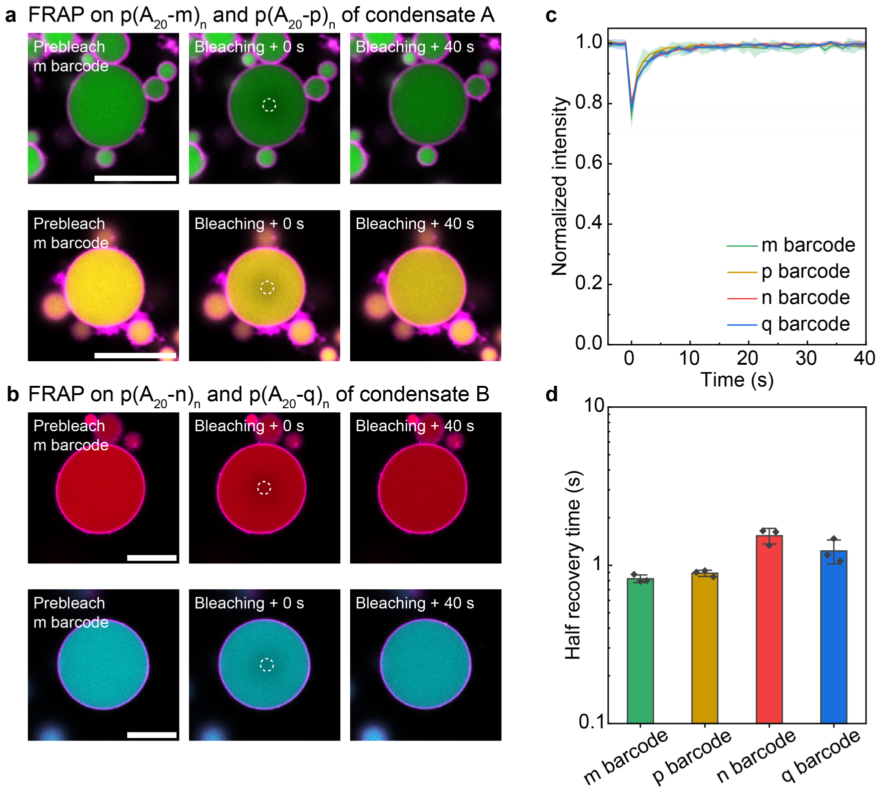

Supplementary Figure 5. Dynamic properties of DNA condensates. (a) Fluorescence recovery after photobleaching (FRAP) experiments on p(A_{20}-m)$_n$ and p(A_{20}-p)$_n$, labeled by Atto488-m* and Atto565-p*, respectively, in a DNA condensate A. (b) Fluorescence recovery after photobleaching (FRAP) experiments on p(A_{20}-n)$_n$ and p(A_{20}-q)$_n$, labeled by Atto565-n* and Atto425-q*, respectively, in a DNA condensate B. (c) Normalized intensity in the bleached areas in (a) and (b), as indicated by white dashed circles, for different DNA polymers inside different condensates, showing fast recovery and very dynamic interior property. (d) Half recovery time ($t_{1/2}$) extracted from the FRAP curves in (c) for different DNA polymers inside different condensates. Shaded areas and error bars represent standard deviation. n = 3 for FRAP experiments. Scale bars are all 10 μm.

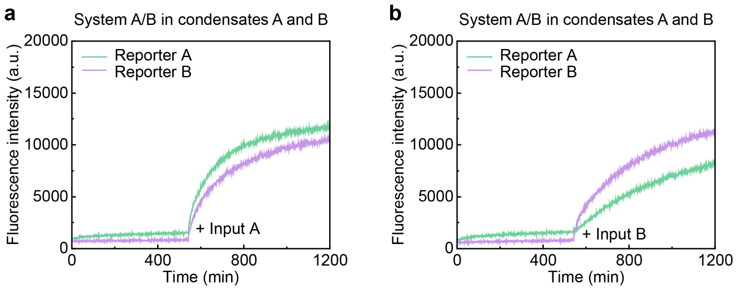

Supplementary Figure 6. When the samples are not washed, the residual DNA duplexes remained in solution impair the control on localized DNA reaction in condensates. (a) When only adding Input A, DNA reaction networks embedded in both condensates A and B will be activated with an increase in both fluorescence signals. Note that the activation of system A is slightly better than the activation of system B due to some marginal level of local reaction for condensate population A. (c) When only adding Input B, DNA reaction networks embedded in both condensates A and B will be activated with an increase in both fluorescence signals. Note that the activation of system A is slightly better than the activation of system B due to some marginal level of local reaction for condensate population B.
